# Hydroxyapatite cement cranioplasty in the setting of simultaneous translabyrinthine resection of cerebellopontine angle tumors and cochlear implantation

**DOI:** 10.3171/2021.7.FOCVID211

**Published:** 2021-10-01

**Authors:** Robert M. Conway, Nathan C. Tu, Pedrom C. Sioshansi, Dennis I. Bojrab, Jeffrey T. Jacob, Seilesh C. Babu

**Affiliations:** 1 Department of Otolaryngology–Head & Neck Surgery, Ascension Macomb-Oakland Hospital, Madison Heights;; 2 Michigan Ear Institute, Farmington Hills; and; 3 Michigan Head & Spine Institute, Southfield, Michigan

**Keywords:** vestibular schwannoma, cochlear implant, cranioplasty

## Abstract

Cochlear implantation (CI) has become an option for the treatment of hearing loss after translabyrinthine resection of vestibular schwannomas. The surgical video presents the case of a 67-year-old male who had translabyrinthine resection of vestibular schwannoma with simultaneous CI and closure with a hydroxyapatite (HA) cement cranioplasty. HA cement cranioplasty can be utilized in place of abdominal fat graft for the closure of translabyrinthine approaches with similar efficacy and complication profile. To the authors’ knowledge, this is the first reported case of a simultaneous CI and translabyrinthine resection of vestibular schwannoma with HA cement cranioplasty.

The video can be found here: https://stream.cadmore.media/r10.3171/2021.7.FOCVID211

## Transcript

The purpose of this video is to demonstrate the surgical technique regarding translabyrinthine resection of vestibular schwannoma with simultaneous cochlear implantation utilizing a hydroxyapatite cement cranioplasty for closure.

Cochlear Corporation supplied a minor number of cochlear implants for the research study that this patient was a part of. There are no direct conflicts of interest with the presented case.

Cochlear implantation is a viable option for hearing rehabilitation when the cochlear nerve is preserved during translabyrinthine resection of the tumor.^1,2^ Cochlear implantation after vestibular schwannoma removal has been performed previously both as a two-stage and single-stage procedure; however, there is limited data on simultaneous implantation.

The typical closure after translabyrinthine resection with cochlear implantation at our institution varies, with both abdominal fat grafting and hydroxyapetite cement having been utilized. HA cement has the benefit of decreased donor site morbidity as well as improved surgical times. However, given the significant cost, cost-effectiveness evaluation is still needed. HA cement is not utilized in CI without translabyrinthine resection at our institution.

### 1:26 Case Presentation.

The surgery presented here is of a 67-year-old male undergoing right-sided translabyrinthine excision of a 1.3 × 1.4–cm vestibular schwannoma. Gross-total resection was performed, and the cochlear nerve was kept intact throughout the surgery.

This patient initially presented to the clinic with sudden hearing loss and tinnitus on the right side. Audiogram of the patient is shown below with a significant right-sided hearing loss with a word recognition score of 40%.

### 1:51 Preoperative Imaging.

Preoperative imaging can be seen here. Depicted is a T1 postcontrast axial and coronal MRI depicting the right-sided vestibular schwannoma.

### 2:01 Surgical Positioning and Incision.

Seen here is the typical surgical positioning with the patient in the supine position and the head turned to the contralateral side. The postauricular incision can be seen here approximately 2 cm from the postauricular crease.

### 2:15 Mastoidectomy.

After the postauricular incision is completed, a typical canal wall-up mastoidectomy is started. The tegmen and sigmoid sinus are decompressed and the dura is begun to be exposed.

### 2:37 Labyrinthectomy.

The labyrinthectomy is then started. In this case, the posterior semicircular canal is taken down initially.

Further drilling of the labyrinth is completed. Seen here is exposure of the superior semicircular canal.

The ampullated ends of the semicircular canals can be seen here along with the common crus and bleeding from the subacute artery.

The completed labyrinthectomy can be seen here with bone overlying the IAC. Posterior fossa dura is seen exposed.

### 3:21 Facial Recess Approach.

Once the majority of the bony labyrinthectomy work is completed, a facial recess approach is utilized to expose the round window.

### 3:30 Internal Auditory Canal Exposure.

Attention is then returned to the remaining bony coverage of the internal auditory canal; a superior and inferior trough is drilled in order to isolate and expose the IAC.

After complete bony removal over the IAC, the dura is incised. Further dissection of the dura was completed, exposing the cerebellopontine angle.

### 4:08 Tumor Removal.

After exposure of the IAC and cerebellopontine angle, the nerve-stimulating spatula instrument is used to ensure that the facial nerve is not on the posterior aspect of the tumor. Once confirmed, tumor debulking is then begun with the intention of finding the facial nerve. Debulking is then continued using an ultrasonic aspirator.

After tumor debulking is completed, the eight cranial nerve is identified proximally in the cerebellopontine angle and tumor dissection is continued from proximal to distal along the nerve, being careful to preserve the overlying blood supply.

Further dissection is completed in the distal aspect of the CPA. Dissection is performed using a stimulating dissector in order to prevent injury to the facial nerve in this location.

As can be seen here, the last segment of tumor is removed and gross-total resection is achieved.

The eighth cranial nerve can be seen here still intact along with the labyrinthine segment of the facial nerve, which is obscured with blood.

After complete resection of the tumor, the orientation is shifted to view the brainstem root entry zone of the facial nerve for final stimulation.

At this point in the surgery, a dural repair substitute was placed into the defect to assist with dural closure and a well and trough was then drilled for the cochlear implant to be placed.

### 6:02 Cochlear Implantation.

A cochleostomy was then performed using a one-diamond burr. In this case, a Cochlear Nucleus Profile Plus with Contour Advance was placed into the cochleostomy site and the electrode is inserted. The stylet is seen being removed here.

Fascia was then used to pack the eustachian tube and around the cochlear implant site; this prevents fistula formation as well as prevents the HA cement from leaking into cochleostomy site.

### 6:54 Closure.

After the cochlear implant is confirmed to be activated with neural response telemetry, closure is begun. Cranioplasty was then performed using hydroxyapatite cement.

In our experience, it is important to hold and maintain pressure immediately after applying the HA cement as this prevents CSF from creating a pathway through the cement prior to the cement hardening.

Of the patients at our institution undergoing concurrent translabyrinthine tumor resection with cochlear implantation, seven have had closure with abdominal fat graft and five had closure with the HA cement. One patient had a CSF leak in the HA cement group; however, this was able to be managed conservatively. A separate patient had a delayed incomplete facial nerve weakness in the HA cement group that is currently being observed.

### 7:40 Postoperative Imaging.

A postoperative T1 postcontrast axial MRI is shown here with gross-total resection being achieved.

### 7:50 Discussion.

Postoperatively, the patient’s CNC testing in the affected ear went from 52% to 34%, demonstrating some viable hearing even after translabyrinthine excision.

His AzBIO testing in both +10 and +5 signal-to-noise ratio improved from his preoperative state, demonstrating an improved ability to hear in background noise after surgery.

To our knowledge, this is the first report of simultaneous translabyrinthine vestibular schwannoma resection and cochlear implantation with hydroxyapatite cement cranioplasty. Hydroxyapatite is a calcium phosphate–based biomaterial with similar properties to bone and teeth that has an isothermic reaction when hardening.^3^ It has been shown that HA cement is a viable alternative to fat grafting with no donor site morbidity and a similar complication profile.^4^ It has been shown to have long-term efficacy and safety with use in translabyrinthine repairs.^5^ A concern with HA cement includes if reexploration of the wound is required.

In the experience of the senior author, HA cement has been easily removed and reapplied for revision cases without cochlear implantation; however, this has not been needed for a case with cochlear implantation to this point.

Simultaneous cochlear implantation with translabyrinthine resection of vestibular schwannoma provides hearing rehabilitation to the patients that would otherwise have profound deafness on the affected side. The demonstrated technique is the authors’ preferred method of surgical steps, and it has been shown that cranioplasty with HA cement is a viable closure option even with cochlear implantation.

## Disclosures

Dr. Babu reports honorarium from Acclarent/Johnson & Johnson and research grants from Cochlear Corporation and Oticon Medical.

Some patients who did not obtain insurance approval for simultaneous cochlear implantation received the implant provided by the cochlear implant company through research support. No financial payment was made to the authors for any part of this study.

## Author Contributions

Primary surgeon: Babu, Bojrab, Jacob. Assistant surgeon: Tu, Sioshansi. Editing and drafting the video and abstract: Conway, Tu, Sioshansi, Bojrab, Jacob. Critically revising the work: Babu, Conway, Tu, Sioshansi, Jacob. Reviewed submitted version of the work: Conway, Tu, Sioshansi, Bojrab, Jacob. Approved the final version of the work on behalf of all authors: Babu. Supervision: Babu, Bojrab.

## Supplemental Information

### Current Affiliations

Dr. Tu: Department of Otolaryngology–Head & Neck Surgery, Albany Medical Center, Albany, NY.

Dr. Sioshansi: Department of Otolaryngology–Head & Neck Surgery, Wake Forest University School of Medicine, Winston-Salem, NC.

## References

[1] Thompson NJ O’Connell BP Brown KD Translabyrinthine excision of vestibular schwannoma with concurrent cochlear implantation: systematic review J Neurol Surg B Skull Base 2019 80 2 187 195 30931227 10.1055/s-0038-1677491 PMC6438800

[2] Bartindale MR Tadokoro KS Kircher ML Cochlear implantation in sporadic vestibular schwannoma: a systematic literature review J Neurol Surg B Skull Base 2019 80 6 632 639 31750050 10.1055/s-0038-1676768 PMC6864424

[3] Ambard AJ Mueninghoff L Calcium phosphate cement: review of mechanical and biological properties J Prosthodont 2006 15 5 321 328 16958734 10.1111/j.1532-849X.2006.00129.x

[4] Luryi AL Schutt CA Michaelides E Kveton JF Hydroxyapatite cement cranioplasty for translabyrinthine surgery: a single institution experience Laryngoscope 2020 130 1 206 211 30843619 10.1002/lary.27907

[5] Volsky PG Hillman TA Stromberg KJ Hydroxyapatite cement cranioplasty following translabyrinthine approach: long-term study of 369 cases Laryngoscope 2017 127 9 2120 2125 28059442 10.1002/lary.26403

